# Spontaneous rupture of intrahepatic pseudocyst into the inferior vena cava

**DOI:** 10.1093/gastro/gow011

**Published:** 2016-04-21

**Authors:** Yashwant Patidar, Binit Sureka, Vaibhav Pratap Singh, Kalpana Bansal, Rakhi Maiwall

**Affiliations:** Department of Radiology/Interventional Radiology, Institute of Liver and Biliary Sciences, New Delhi, India

**Keywords:** pancreatic pseudocyst, spontaneous rupture, inferior vena cava

## Abstract

We present an extremely rare case of caudate lobe intrahepatic pancreatic pseudocyst with spontaneous rupture into the inferior vena cava (IVC). A 58-year-old male, a chronic alcoholic, presented with features of pancreatitis for which imaging was done. Ultrasound and contrast-enhanced computed tomography were carried out, which revealed intrahepatic pancreatic pseudocyst in the caudate lobe of the liver. There was suggestion of spontaneous rupture of the pseudocyst into the IVC, which was well delineated on imaging.

## INTRODUCTION

Pancreatic pseudocyst is a common complication of acute interstitial oedematous pancreatitis. Pseudocyst is defined as an encapsulated collection with a well defined inflammatory wall, usually outside the pancreas, without evidence of any necrosis [1, 2]. These are fluid-filled collections that have a non-epithelialized wall consisting of fibrous and granulation tissue. This entity usually occurs more than 4 weeks after the initial acute episode. There can be multiple occurrences, which can be found anywhere from the groin to the mediastinum. The location of a pseudocyst in the liver is an exceptional event and only 33 cases had been reported in the literature up to 2009 [3]. Major vascular complications of pancreatitis are also well known, occurring with a frequency of 1.2–14% [4]. Vascular complications occurring secondary to a pseudocyst may involve the arterial circulation in the form of haemorrhage or pseudoaneurysm, or the venous system in the form of thrombus, rupture or fistulisation involving the splenoportal system [5]. We report an extremely rare vascular complication of intrahepatic pancreatic pseudocyst rupturing into the inferior vena cava (IVC).

## CASE PRESENTATION

**Figure 1.**
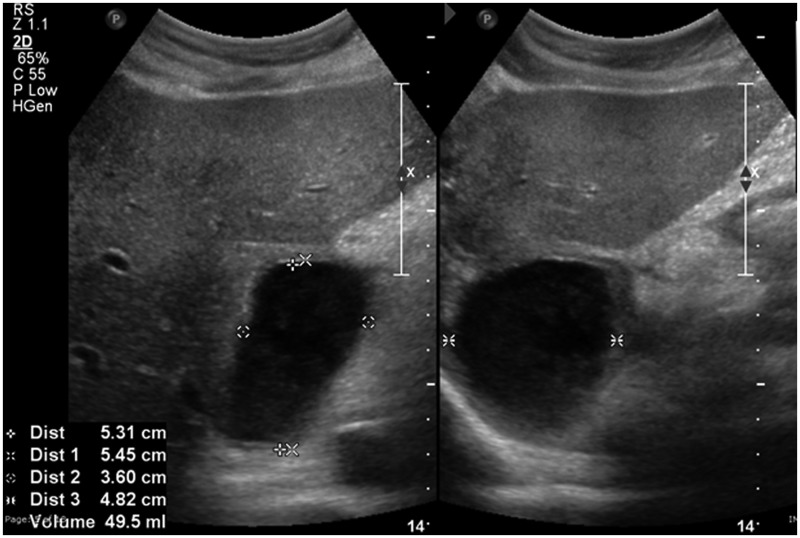
Ultrasound image showing pseudocyst in the caudate lobe

**Figure 2.**
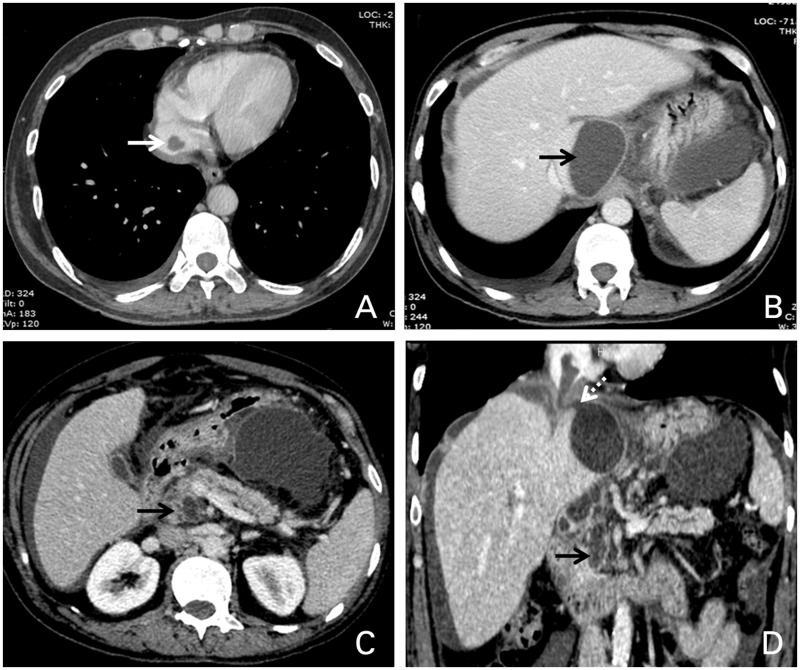
Axial contrast-enhanced CT image showing (A) pseudocyst rupture into the suprahepatic IVC; (B) caudate lobe pseudocyst; (C) pseudocyst in the pancreas. (D) Coronal contrast-enhanced CT image showing well-delineated track (dotted arrow) and pancreatic pseudocyst (arrow).

A 58-year-old male, a chronic alcoholic, presented to us with mild-to-moderate abdominal pain in the epigastric region, with presence of fever for the previous 10 days, for which he was admitted in our institute for further evaluation and management. Ultrasonography (USG) of the abdomen was done in the emergency setting, revealing chronic liver parenchymal disease with mild ascites and multiple collections in the abdomen, one of which was in the caudate lobe of the liver (Figure 1). The patient showed elevated serum amylase (674 U/L) and serum lipase (4897 U/L). Thereafter contrast-enhanced computed tomography (CT) of the whole abdomen was carried out. The patient’s CT scan confirmed multiple collections in the abdomen. The unusual thing was a large intrahepatic collection in the caudate lobe, approximately 3.7 x 4.8 x 5.8 cm. The caudate lobe collection in its superior aspect was communicating with the IVC through a narrow tract approximately 5 mm in thickness and extending superiorly as far as the right atrium with an average attenuation value of 15–30 HU (Figure 2). Apart from the intraluminal IVC extension, the caudate lobe collection was also seen extending and communicating inferiorly with the peripancreatic collection near the head of the pancreas. The collection showed few internal septations. Collections were also seen in the lesser sac, mesentery and perigastric region.

The patient was managed conservatively. On the day following screening ultrasound, there was a significant reduction in the size of the caudate lobe collection. The patient had no fever or signs of septicaemia, so conservative management was continued and the patient was discharged after 5 days.

## DISCUSSION

Formation of pseudocysts is a well-known and frequently diagnosed complication of pancreatitis. The most common location of a pseudocyst is in the lesser sac, in close proximity to the antero-superior border of the pancreas. Large pseudocysts can extend into the paracolic gutters, pelvis, scrotum, mediastinum or adjacent spleen. A less common but potentially lethal complication is the communication of a pancreatic pseudocyst with adjacent vessels. Fistulisation into the portal vein is a rare consequence of pancreatic pseudocyst formation and only 18 cases have been reported [6].

Occasional case reports of pancreatitis associated with IVC thrombosis have been described in the literature but rupture of intrahepatic pseudocyst into the IVC is an extremely rare entity. The mechanism by which the rupture/fistula forms is poorly understood. It is well known that pancreatic enzymes—including lipase, amylase and other proteolytic enzyme—are present in high concentrations in pseudocysts. High concentrations of pancreatic enzymes within a pseudocyst can be responsible for its invasion into adjacent structures [7]. Thrombosis may result from mass effect and compression by the pseudocyst, along with associated peri-pseudocyst inflammation [8]. Alternatively, fistulisation and release of digestive enzymes into an adjacent vessel may directly cause intravascular thrombosis [9].

Serious complications seen with the fistulisation of the pancreatic pseudocyst-portal vein are septic shock, systemic inflammation, lipolysis and haemorrhage [10]. Similar complications can be anticipated in cases of pseudocyst rupturing into the IVC. Our patient initially had a fever, which may have been due to the rupture. Thereafter he had an uneventful course at our institute and was managed conservatively with supportive treatment. In our case, the pseudocyst disappeared quickly due to self-drainage of the sterile fluid into the adjacent vascular system, i.e. the IVC. This could be presumed to be a self-regulatory and self-healing mechanism of the human body.

No specific recommendations are available for the management of such unusual cases; however a multidisciplinary team approach is usually advisable, with decompression of symptomatic pseudocysts and walled-off collections by drainage. The drainage can be done by percutaneous, endoscopic, or surgical techniques. Caution must be exercised if there is a pseudoaneurysm within the collection, due to the risk of bleeding. The pseudoaneurysm must first be taken care of by embolization and thereafter the drainage procedure may be carried out. Future reports will help us in framing possible aetiopathogenesis, clinical progression and management guidelines.

*Conflict of interest statement*: none declared.
